# Synchrotron-based X-ray fluorescence microscopy enables multiscale spatial visualization of ions involved in fungal lignocellulose deconstruction

**DOI:** 10.1038/srep41798

**Published:** 2017-01-31

**Authors:** Grant Kirker, Sam Zelinka, Sophie-Charlotte Gleber, David Vine, Lydia Finney, Si Chen, Young Pyo Hong, Omar Uyarte, Stefan Vogt, Jody Jellison, Barry Goodell, Joseph E. Jakes

**Affiliations:** 1 Durability and Wood Protection Research, USDA Forest Service, Forest Products Laboratory, Madison, WI, USA; 2 Building and Fire Sciences, USDA Forest Service, Forest Products Laboratory, Madison, WI, USA; 3 Advanced Photon Source, Argonne National Laboratory, Argonne, IL, USA; 4 Department of Physics and Astronomy, Northwestern University, Evanston, IL, USA; 5 Departamento de Biotecnologia, Escola de Engenharia de Lorena, Universidade de São Paulo, Brazil; 6 Centro I+D, CMPC Celulosa, Chile; 7 Center for Agriculture, Food and the Environment, University of Massachusetts - Amherst, Amherst, MA, USA; 8 Department of Microbiology, University of Massachusetts - Amherst, Amherst, MA, USA; 9 Forest Biopolymers Science and Engineering, USDA Forest Service, Forest Products Laboratory, Madison, WI, USA

## Abstract

The role of ions in the fungal decay process of lignocellulose biomaterials, and more broadly fungal metabolism, has implications for diverse research disciplines ranging from plant pathology and forest ecology, to carbon sequestration. Despite the importance of ions in fungal decay mechanisms, the spatial distribution and quantification of ions in lignocellulosic cell walls and fungal hyphae during decay is not known. Here we employ synchrotron-based X-ray fluorescence microscopy (XFM) to map and quantify physiologically relevant ions, such as K, Ca, Mn, Fe, and Zn, in wood being decayed by the model brown rot fungus *Serpula lacrymans*. Two-dimensional XFM maps were obtained to study the ion spatial distributions from mm to submicron length scales in wood, fungal hyphae with the dried extracellular matrix (ECM) from the fungus, and Ca oxalate crystals. Three-dimensional ion volume reconstructions were also acquired of wood cell walls and hyphae with ECM. Results show that the fungus actively transports some ions, such as Fe, into the wood and controls the distribution of ions at both the bulk wood and cell wall length scales. These measurements provide new insights into the movement of ions during decay and illustrate how synchrotron-based XFM is uniquely suited study these ions.

Understanding the role of ions in fungal degradation of woody biomass is salient to a wide range of research disciplines including carbon sequestration^1^,^2^, forest ecology^3^,^4^, plant pathology^5^, biorefinery^6^,^7^, basic fungal physiology^8^, and the durability and utility of wood in structural applications^9^,^10^,^11^,^12^,^13^. It is estimated that 10% of the lumber harvested each year is used to replace in-service wood that has been attacked by decay fungi^14^. Nearly all structural lumber in the northern hemisphere is comprised of softwoods (conifers, gymnosperms); these wood species are especially susceptible to attack from brown rot fungi. Ion transport related to biomimicry of brown rot is also of particular interest in developments to improve the efficiency of processing biomass for platform chemical production^6^,^7^. Brown rot is a specialized mode of wood decay employed by approximately 6% of the species of wood rotting fungi. Mechanisms of brown rot are not fully understood, but have been shown to involve a complex sequence of reactions including mediated redox chemistry, chelation and reduction of metals, and acidification of the substrate^15^,^16^. The goal of this work was to utilize two- and three-dimensional X-ray fluorescence microscopy (XFM) ion mapping to study physiologically relevant ions during wood biodegradation by examining a model brown rot fungus.

Classic brown rot is typically initiated when hyphae come into contact with the cell lumen in the presence of moisture and produce extracellular H_2_O_2_ to initiate production of oxygen radicals via a chelator-mediated Fenton (CMF) reaction that permeates the lignocellulose cell wall, and weakens the cellulose matrix^17^. Excess iron and other transition metals in the decay microenvironment also help to regulate the production of low molecular weight fungal chelators, such as oxalate, that sequester transition metals, or hydroquinones that sequester iron from oxalate and then subsequently reduce it to participate in Fenton reactions^17^,^18^. From an applied standpoint, mimicking the cation redox activity that occurs during incipient decay may lead to improved biorefinery pretreatments, and arresting cation redox activity may lead potentially to better ways of sequestering carbon, or protecting wood products from decay.

Studies of the roles that ions play in decay processes have relied largely on bulk measurements of the ion concentrations of decayed wood, which includes ion contributions from both degraded wood and fungal hyphae. But these analyses do not distinguish between localization of ions within the cell wall versus within the lumen. Most have involved grinding or other disruption of wood, which also disrupts localized concentrations of the ions. Jellison *et al*.^9^ reported that concentrations of Ca, Mn, and Fe were higher in wood decayed by various fungi. They concluded that the ability of fungi to modify their ionic environment is of potential importance in their ability to colonize and degrade wood. Ostrofsky *et al*.^10^ reported that Ca and K tended to increase linearly with fungal exposure in red spruce. Green *et al*.^11^ used chrome azurol and inductively coupled plasma analysis to measure increased metal cations in decaying wood by various brown and white rot fungi and found increased Fe and possibly Al in brown rotted wood. Further, Daniel, Blanchette and others were able to localize manganese in white rotted wood, presumably associated with white rot enzymatic attack^19^,^20^,^21^.

Illman and Bajt^12^ analyzed redox states of Fe using synchrotron-based X-ray absorption near edge spectroscopy showing increased Fe^2+^/Fe^3+^ ratios during brown rot decay, while related work with electron paramagnetic resonance in the same paper showed a 10x increase in Mn during decay (Fe levels were not assessed). Jellison *et al*.^13^ reported that Ca, Mn, and Fe fluctuate in a model brown rot fungus and may provide a way for fungi to translocate macro and micronutrients bi-directionally throughout the process of decay. Schilling and Jellison^22^ have shown that the brown rot fungus *Gloeophyllum trabeum* can translocate Ca from drywall made from gypsum into the decaying substrate in building applications, but that metal concentrations including K, P, Fe, Mn, Na, and Zn in wood decayed by four brown rot fungi were not significantly different from undecayed wood. Soil has generally been found to contribute the majority of the metals translocated into wood and an increasing concentration of metals is often associated with increased decay related weight loss. These studies highlight the importance of ion translocation in the decay process, but bulk measurements provide no information about the spatial distribution of the ions in decaying wood. Furthermore, they cannot distinguish between ions inside the fungal hyphae and wood cell walls.

X-ray fluorescence microscopy (XFM) using synchrotron sources is capable of mapping trace amounts of elements in decaying wood across multiple length scales down to the sub-micron spatial resolution needed to probe individual wood cell walls and fungal hyphae. In XFM, X-rays are monochromatized to energy above the absorption edges of the elements of interest and focused onto the sample. The incident X-ray can be absorbed by an atom, which leads to the ejection of an inner-shell electron leaving the atom in an excited state. For medium- to high-Z elements, the inner-shell vacancy is usually filled by the relaxation of an outer-shell electron and excess energy is given off by a fluorescence photon with an energy characteristic of the element. Fluorescence photons are detected by an energy dispersive detector and the type and amount of element present in the sample can be determined with very high sensitivity. By raster scanning the focused beam over the sample, maps of numerous elements can be made simultaneously^23^,^24^,^25^. Recent developments have used the high spatial and concentration resolution of XFM to study the moisture-induced diffusion of ions in wood cell walls^26^ and the infiltration of Br-labeled adhesives into wood cell walls^27^. Here we present measurements showing how XFM can be employed to understand the use and movement of physiologically relevant ions by wood decay fungi. Synchrotron-based XFM was used to investigate ions in wood degraded by a brown rot fungus, *Serpula lacrymans*, across multiple length scales, including the bulk wood level, locally within wood cell walls and also within and surrounding individual fungal hyphae. *Serpula lacrymans* was chosen because it is a well-studied model brown rot wood decay fungus that is economically important.

## Results

**Figure 1:**
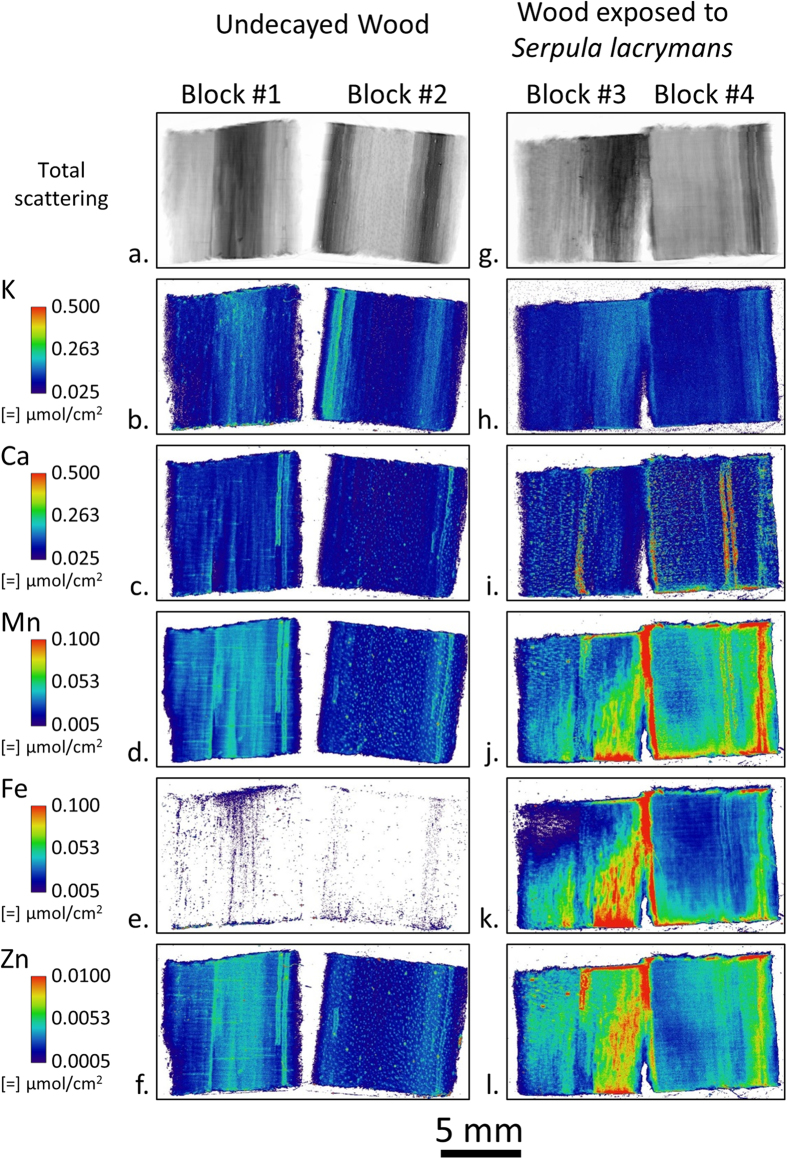
Large field-of-view XFM ion maps of ion distribution in undecayed wood blocks (**a**–**f**) and wood blocks exposed to the model brown rot fungus *Serpula lacrymans* for 14 days in a soil block test (**g**–**l**). Two blocks were imaged for each treatment. The longitudinal wood axis was oriented vertically and the bottoms of the exposed blocks were in contact with the feeder strip during the soil block test. For a given ion, the intensities were scaled the same between blocks. Between ions, intensities were scaled to optimize visualization of ion spatial distribution.

Figure 1 shows the two-dimensional spatial distributions of K, Ca, Mn, Fe, and Zn ions in undecayed southern pine (*Pinus* spp.) wood (Blocks 1 and 2) and wood exposed to *Serpula lacrymans* in a soil-block test for 14 days (Blocks 3 and 4). The undecayed wood blocks were taken from the same board as the infected samples, but before fungal inoculation and sterilization. Ion maps were collected by cleaving an approximately 2 mm thick longitudinal section from the center of each wood block using a carbon steel razor blade and then raster scanning the block surfaces with an approximately 25 μm diameter X-ray beam. Undecayed wood samples confirmed that limited to no transfer of metal from the razor blade occurred. Because of the penetrating nature of hard X-rays, which reach several hundred microns into the surface of wood samples, the ion maps produced represent a 2D area projection of a 3D volumetric measurement and are reported in units of moles per area. Therefore, the ion maps include contributions from both the wood cell walls and any hyphae colonizing the cell lumina because the beam interaction volume would be large enough to include them. However, because the beam interaction volume is only approximately known, the amount of ions per unit volume of material, or ion concentration, cannot be calculated for these XFM maps. Nevertheless, ion maps like these in Fig. 1 are useful for comparing relative amounts of different ions in the wood and the effect of decay on ion spatial distributions across the wood block. Additionally, molar intensities were chosen instead of mass intensities to facilitate more direct comparisons of XFM maps with the conventional techniques used to quantify ions in bulk wood, like inductively coupled plasma spectroscopy, which are reported in molar quantities. Moreover, when considering chemical reactions like the CMF reaction, it is the number of ions and not the mass of ions that is more relevant to understanding the reactions.

The total XFM scattering maps (Fig. 1a and g) permitted visualization of the wood section and in these maps the darker regions corresponded to latewood growth rings. In the undecayed wood block ion maps (Fig. 1b–f), differences in concentrations and spatial distribution of ions corresponded to wood anatomical features, such as the increased concentration of K in the latewood regions. Additionally, visual inspection revealed that the vertical feature on the right-hand side of Block 1 highlighted by the increased concentrations of Ca, Mn, and Zn (Fig. 1c,d and f, respectively) that extends from the top of the block and abruptly ends three fourths of the way to the bottom, corresponded to a resin canal. In contrast, in the wood blocks exposed to *Serpula lacrymans*, some distributions of ions did not correspond to wood anatomical features and were presumably controlled by the fungus colonizing the wood block. For instance, in Block 3, diagonal gradients in Mn, Fe, and Zn ion maps (Fig. 1j–l, respectively) were observed with the lower right-hand corner of the block having substantially increased concentrations. The highest concentrations of these ions were nearest the bottom of the block, which was in direct contact with the feeder strip. The differences within a block likely represent different stages of fungal colonization and decay within the block. Both Blocks 3 and 4 were placed on the same feeder strip for the same amount of time. The differences in Mn, Fe, and Zn maps between these blocks suggest differences in the rate and progression of the decay of these two blocks. Interestingly, the overall distribution of K, a highly mobile cation in aqueous environments, did not change after exposure. In Ca, there was an increase in localized areas of concentration (hotspots), but the overall background intensity did not change.

**Figure 2:**
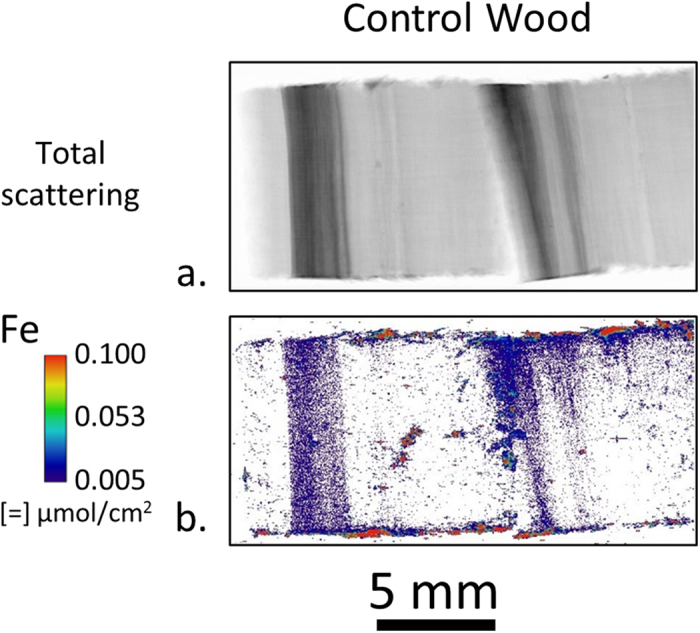
Large field-of-view XFM ion maps of Fe distribution in two sterilized control wood blocks placed directly on top of the soil for 8 weeks in a soil block test bottle. During the 8 weeks the blocks inadvertently rolled around on top of the soil and the areas of high Fe intensity are likely small pieces of soil. However, no general diffusion of Fe into the wood is observed like that observed in wood blocks exposed to *Serpula lacrymans* in Fig. 1k.

A prominent difference between the undecayed wood and wood exposed to *Serpula lacrymans* is the increase in Fe concentration after exposure. Although introduction of Fe into the samples during sterilization or passive diffusion of soil Fe through the feeder strip and into the test block was plausible, we examined these possibilities in appropriate control experiments. For these controls, a sterilized test block was placed directly onto the soil in a soil-block test without fungal inoculation for 8 weeks and a center section was cleaved and imaged the same way using XFM. The results showed no Fe diffusion into the control test block (Fig. 2). Therefore, *Serpula lacrymans* must have actively solubilized soil Fe and transported Fe from the soil and into Blocks #3 and 4 in Fig. 1. Once in the test block, the fungus also controlled where the Fe was distributed as observed on a mm length scale. Generally, regions of higher Fe concentration also had higher amounts of Mn and Zn. In the natural environment, iron is found in insoluble, oxidized states^28^,^29^,^30^ and to solubilize and mobilize iron, microorganisms use low molecular weight metal binding compounds^31^,^32^. The observed solubilization and translocation of metal cations is consistent with the ability of brown rot fungi, such as *Serpula spp*. to both reduce the pH of their environment and to produce effective metal solubilization and chelating compounds^15^,^33^.

**Figure 3:**
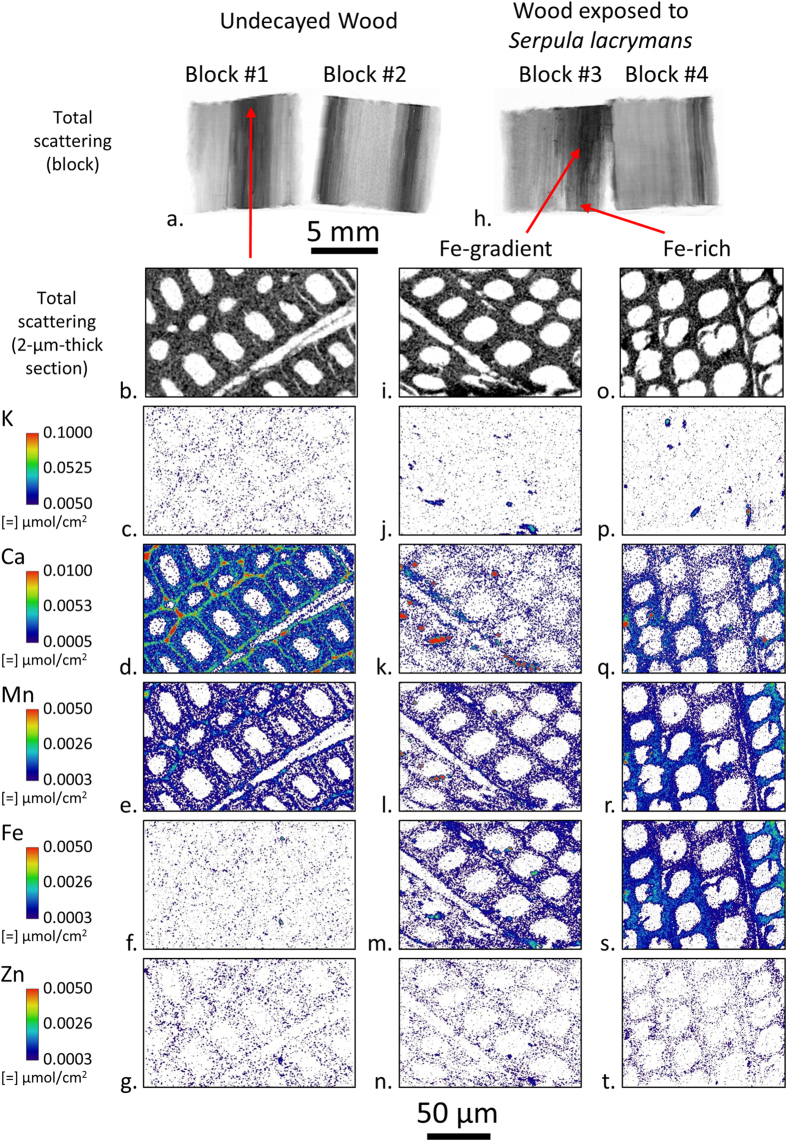
Submicron resolution XFM ion maps of 2-μm-thick transverse sections cut from undecayed wood Block 1 and exposed Block 3 in Fig. 1. For a given element, the intensities were scaled the same between sections. Between elements, intensities were scaled to optimize ion distribution visualization.

To investigate ion distribution in individual wood cell wall layers, 2-μm-thick transverse sections were cut from latewood in the undecayed wood Block 1, the Fe-rich region near the bottom of Block 3, and the Fe-gradient area of Block 3 as shown in Fig. 3. Latewood was chosen instead of earlywood for sectioning because the thicker cell walls made it easier to prepare intact 2-μm-thick sections. The sections were cut near the surfaces that were scanned to facilitate direct comparison with the large field-of-view ion maps in Fig. 1. The sections were held flat between two 200 nm thick SiN windows and an approximately 0.5 μm diameter X-ray probe was raster scanned over the sections to build the ion maps. Again, total scattering XFM maps were used to aid visualization of the cell walls. The thin sections and submicron X-ray spot size made it possible to distinguish individual cell wall layers, as exemplified by the increased concentration of Ca in the middle lamellae as compared to the secondary cell walls in the undecayed wood (Fig. 3d). Hotspots could also be observed in the K (Fig. 3j and p) and Ca (Fig. 3k and q) maps in the exposed block sections. These hotspots may be dried extracellular matrix (ECM) material (primarily ß-glucans in an aqueous hydrogel) or dried ECM with included hyphae that were cut from hyphae colonizing the lumina of these cells during section preparation. The drying process would have caused the ECM to shrink from its original position surrounding the fungal hyphae, and also concentrated ions that would have been present in the hydrated ECM. The ECM is known to connect the fungal hyphae to the wood cell wall during decay, but when drying occurs the ephemeral nature of the ECM sheath causes it to shrink and deposit in locations along the fungal hyphae or the wood cell wall where it was originally attached.

The concentration of Fe in the wood cell walls increased substantially after exposure to the fungus (Fig. 3m and s) as compared to the undecayed wood (Fig. 3f). In the Fe-rich section (Fig. 3s), there is an inhomogeneous Fe distribution with regions of increased Fe concentration in the lower left-hand and upper right-hand regions of the Fe map. Additionally, Ca, Mn, and possibly Zn are co-localized with these regions of increased Fe intensity in the section (Fig. 3q–t). Based on our previous work examining ion gradients in wood under high humidity conditions^26^, these gradients would not be expected if only passive diffusion controlled ion transport in the wood cell walls. This suggests that the fungus actively controls the local microenvironment at the cellular length scale in such a way that results in an inhomogeneous ion distribution. It is known that *Serpula lacrymans* and other brown rot fungi reduce the pH of the environment immediately surrounding the fungal hyphae (within the ECM), and that in combination with the secretion of calcium-oxalate, as well as, redox-cycling chelators helps to solubilize and reduce iron and perhaps other transition metals^15^,^33^. Oxalate would sequester Fe and other transition metals, exchanging those metals for Ca, and we posit that this would result in the potential co-localization with Fe of both other transition metals and Ca within the ECM. These reactions are known to occur during initiation of redox cycling by catecholate chelators in CMF reactions, and co-localization of Ca and transition metals may be indicative of sites of decay initiation. Oxalate has been reported to diffuse in very low concentrations in the wood cell wall even after wood is incubated with high concentrations of oxalate, and then exposed to brown rot fungal attack^34^. This suggests that once oxalate transfers its Fe to redox cycling chelators and these diffuse into the cell wall, the Ca in the wood cell wall would diffuse out, in part to complex with oxalate in the lumen.

The ion intensities in the XFM maps in Fig. 3 can be used to calculate volumetric ion concentrations in the wood cell walls because the sections have well-defined 2-μm thicknesses and were thin enough for the X-ray beam to completely penetrate. The ion intensities in Fig. 3 were converted to μmoles/g cell wall as shown in Table 1. The quantified concentrations confirmed the visual observations in Fig. 3 that there was relatively little change in the overall K, Mn, and Zn cell wall concentrations after exposure to *Serpula lacrymans*. The Fe increased in the Fe-gradient region compared to the undecayed wood and on average increased even more in the Fe-rich region. Interestingly, a substantial decrease in Ca within the cell wall was observed after decay compared to the undecayed cell walls, which supports our hypothesis outlined above on the exchange of Ca for Fe in oxalate. The quantified ion concentrations we calculated can also be used to help compare XFM results to results from conventional techniques like the inductively coupled plasma spectroscopy used in previous studies to quantify bulk μmoles/g wood ion concentrations during decay^9^,^10^,^35^. Comparatively, XFM cell wall ion concentrations are within an order of magnitude of the conventional bulk measurements, and this indicates the two methods are in alignment considering the differences in wood, fungi used, overall experimental design in the different studies and that the bulk measurements include ions in both the wood and hyphae colonizing the wood. The major advantage of the XFM measurement is that it allows a direct localized quantification of ion concentrations in wood cell walls and lumens.

**Figure 4:**
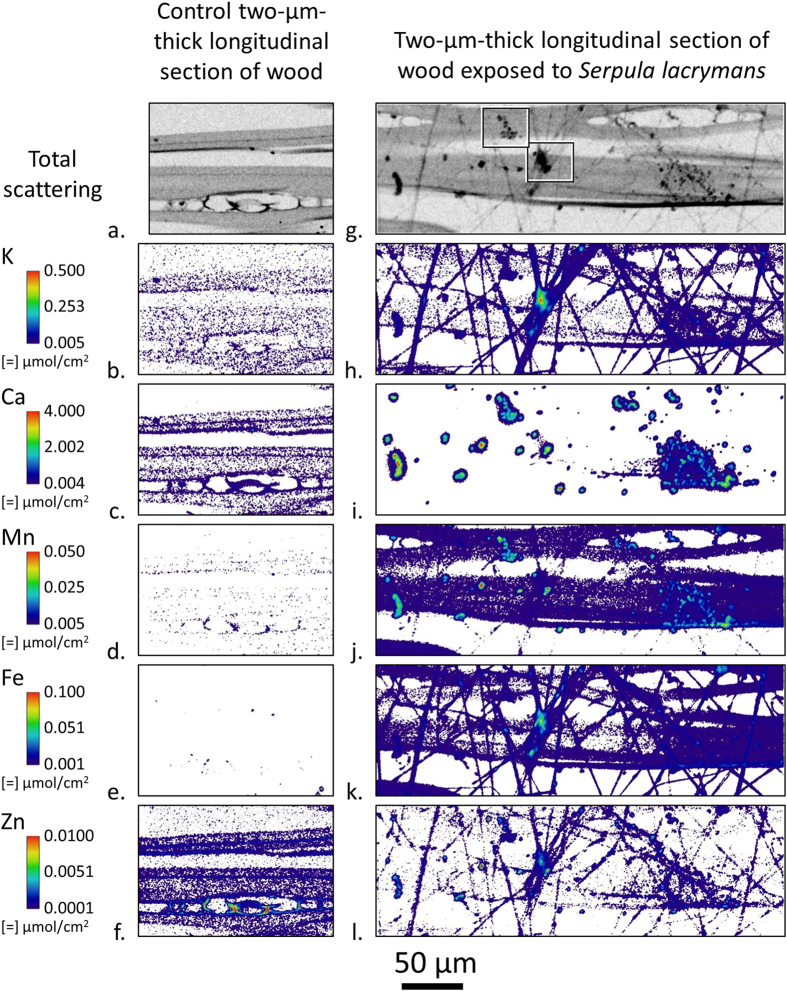
Submicron resolution XFM ion maps of a 2-μm-thick tangential-longitudinal section exposed in a control soil block chamber (**a**–**f**), and a *Serpula lacrymans* inoculated feeder strip in a soil block test for 10 days (**g**–**l**). For a given element, the intensities were scaled the same between sections. Between elements, intensities were scaled to optimize visualization of ion distribution. The threadlike features in the exposed total scattering map (**g**) were hyphae. The amorphous bodies (most obvious in 3i) which show colocation of Ca with other ions around the fungal hyphae were putative ECM which has dried around the fungal hyphae and concentrated ions that originally would have been distributed throughout the hydrated ECM matrix connecting the fungal hyphae to the wood cell wall. The boxes in the exposed total scattering map (**g**) highlight regions for ion colocation maps in Fig. 6.

To study intact fungal hyphae, 2-μm-thick tangential-longitudinal sections were exposed directly to *Serpula lacrymans* inoculated feeder strips in the soil block test. After fungal exposure, the sections were carefully removed from the feeder strip and two-dimensional ion maps of both the wood cell walls and fungus were made using a 0.5 μm diameter X-ray probe (Fig. 4g–l). Additionally, a control section was exposed to a feeder strip inside of a sterile soil bottle without fungal inoculation, and ions were mapped inside the wood cell walls (Fig. 4a–f). Compared to the control wood cell walls, the concentrations internal to the cell walls of Ca and Zn decreased while Mn and Fe increased after fungal exposure. These changes show the fungus interacted with the 2-μm-thick wood section and again clearly demonstrates the fungus actively transports ions, such as Fe, into and within the sections.

In the ion maps, hyphae are distinctly associated with concentrations of K and Fe with some Zn and Mn. In Fig. 4i, the Ca intensity is scaled to better show the numerous localized hotspots on the surface of the cell wall and surrounding fungal hyphae, but Ca could also be detected intermittently along the hyphae. Observation of the exposed section using an optical microscope revealed that not all of the hyphae were lying on the section surface and that some were suspended above and below the section. Again, these ion maps are 2D projections of the 3D X-ray beam interaction volume, and differences in ion intensities could be caused by differences in the amount of material in the interaction volume. However, for a given location these maps provide accurate comparisons of the relative amounts of ions.

**Figure 5:**
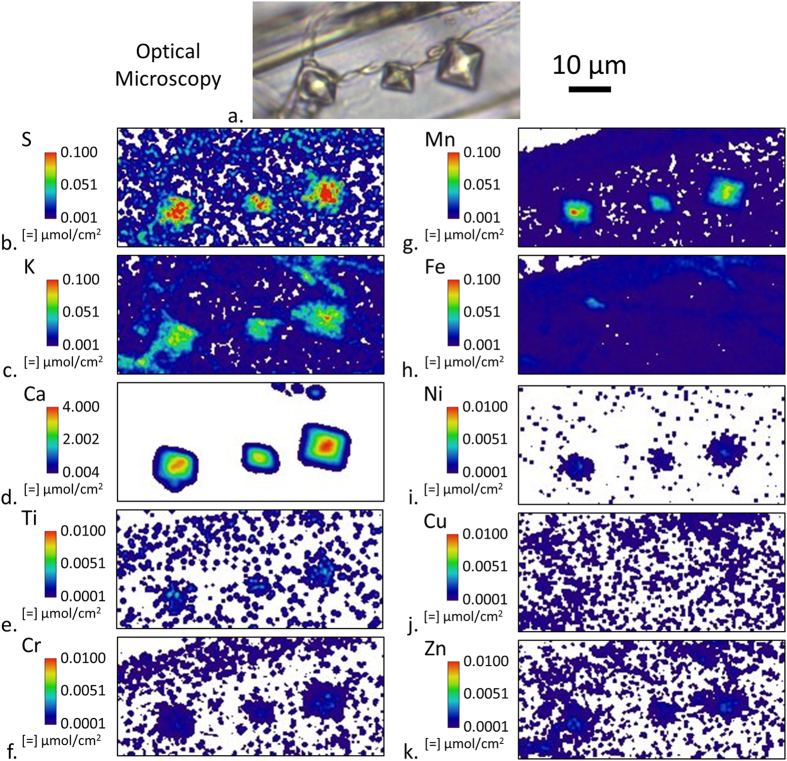
Optical microscopy (**a**) and submicron resolution XFM ion maps of three oxalate crystals within a 2-μm-thick tangential-longitudinal section that was exposed to a *Serpula lacrymans* inoculated feeder strip in a soil block test for 20 days. Between elements, intensities were scaled to optimize visualization of ion distribution.

Many of the Ca hotspots in Fig. 4c are likely insoluble calcium-oxalate crystals within the residual ECM. Figure 5 shows three definitive crystals as observed by both optical microscopy and XFM. In addition to Ca, the elements S, K, and Mn are also detected in the crystals and the XFM quantification suggests that the crystals are approximately 2% S, K, and Mn on a molar basis. Trace amounts of Ti, Cr, Ni, and Zn were also detected in the crystals. However, no Fe or Cu was detected as Fe or Cu would only be exchanged in the soluble forms of oxalate.

The Ca concentration in the calcium-oxalate crystals in Fig. 5 was also estimated to further assess the accuracy of XFM against the known molar concentration of Ca in these crystals. The octahedral crystal shapes are indicative of the Ca being in a weddelite crystal structure whose chemical formula is CaC2O_4_·2(H_2_O), of which Ca constitutes 24.4% of the mass^36^. Given that weddellite has a density of 2.02 g cm^−3^, the expected Ca concentration in an oxalate crystal would be 0.5 g cm^−3^. For each of the three crystals in Fig. 5d, the average molar intensity was assessed and then converted to mass intensity using the molecular weight of Ca. The experimental amount of Ca in each oxalate crystal was then estimated by assuming the amount of Ca in the cell wall was negligible and multiplying the average Ca intensity in each crystal by its area occupied in the XFM map. The volume of the crystal was estimated by assuming the crystal shape was a regular octahedron with a volume equal to 0.471*a*^3^ where *a* is the edge length measured from the XFM map. Finally, the Ca concentration was estimated by dividing the measured amount of Ca in each crystal by its calculated volume. Using this procedure, values of 0.38, 0.53, and 0.33 g cm^−3^ (from left to right, respectively) were calculated for the crystals’ Ca concentrations. The average 0.4 g cm^−3^ Ca concentration measured compared very well to the theoretical 0.5 g cm^−3^ value considering the uncertainty of the actual crystal shape and the amount of other ions (e.g. Mn) observed in the crystal that are likely occupying Ca sites on the crystal lattice. This data helps to provide confirmation of the usefulness and accuracy of XFM in calculating other ion concentrations within the wood cell wall.

**Figure 6:**
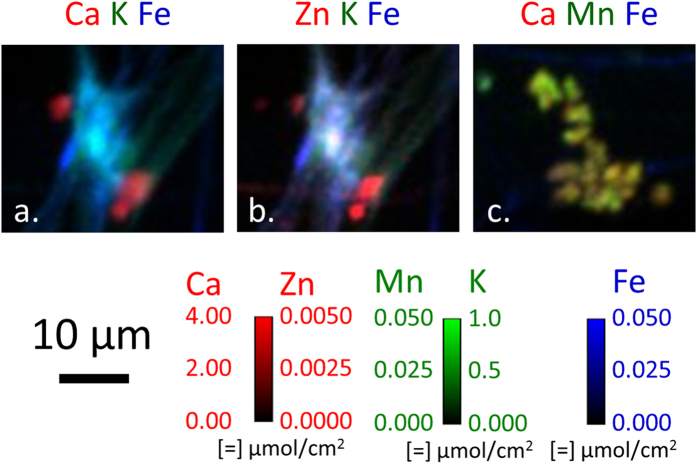
2D ion colocation maps of Ca K Fe (**a**) and Zn K Fe (**b**) of a putative hyphal anastomosis in the area of the right-hand box drawn in Fig. 4g. Ca Mn Fe (**c**) colocation map of putative dried ECM from the left-hand box. Color intensities were scaled to optimize spatial visualization of ion distribution.

**Figure 7:**
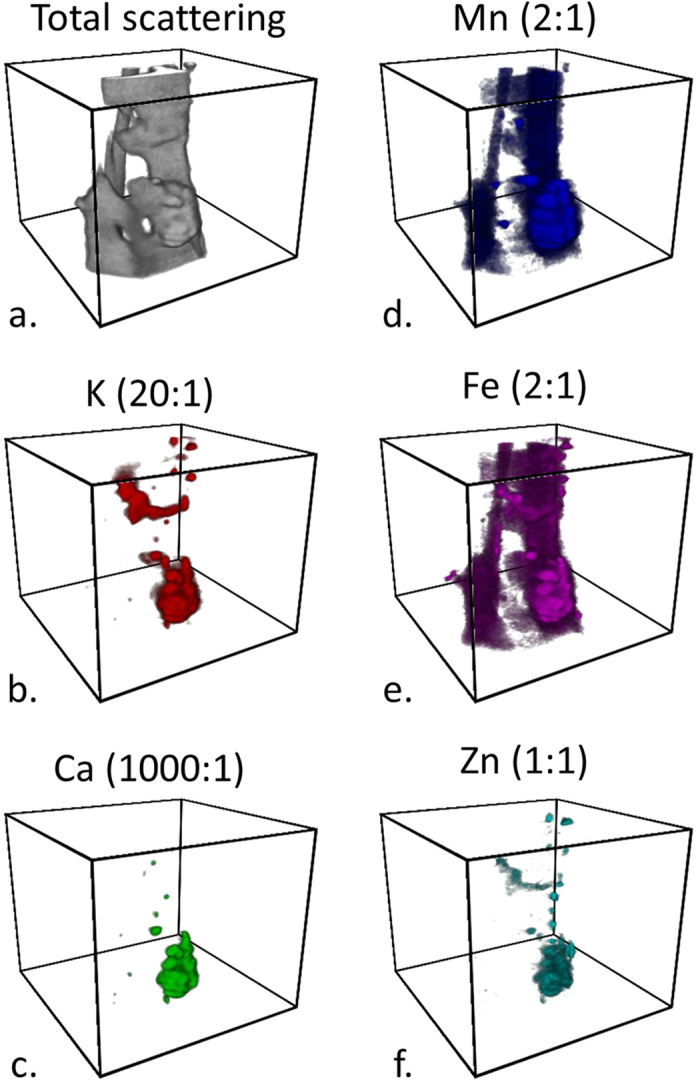
Micron-scale resolution XFM ion volume reconstructions of a wood sliver and attached fungal fragments and ECM residue cut from the Fe-rich region near the bottom of exposed Block 3 (Fig. 1). During tomographic reconstruction, the quantification of the ions was lost, however relative ion concentration differences can be detected and compared. The ratios indicated are molar ratios with respect to the amount of Zn. The boxes have a square base of 90 μm per side and a 75 μm height. See Supplementary Movie 1 for 360 degree rotations of volumes.

Concentrations of K, Fe, and Zn in Fig. 4h,k and l, respectively, appear to be located in areas of hyphal intersections, or possible areas of anastomosis. These areas are typically associated with an accumulation of ECM and colocation of ions is seen in this area. Although the ECM is dried and no longer in its native form, 2D ion colocation maps are useful to study ion spatial distributions and relative amounts of ions. Ca K Fe and Zn K Fe ion colocations in these zones are shown in Fig. 6a and b, respectively. In the Ca K Fe colocation, distinct Ca concentrations, which are likely oxalate crystals, are observed on the upper left and lower right hand corners just outside of the K- and Fe-rich region highlighting the ionic cluster. The Zn K Fe colocation shows inhomogeneous Zn and Fe distribution within this region, but given the shrinkage of the ECM which occurred, redistribution of ions would be expected. In the center of this region there is a Zn hotspot measuring 2–3 μm across. Figure 7c shows a Ca Mn Fe colocation of another commonly observed ion concentration region that is likely dried ECM.

XFM tomography can also be performed with nearly the same spatial resolution and sensitivity as the 2D XFM mapping to study distributions of ions in 3D. To study fungi in decayed wood using XFM tomography, an infected wood sliver approximately one tracheid-width across was excised from the Fe-rich region near the bottom of Block 3 in Fig. 1. Figure 7 and Supplementary Movie 1 show ion volume reconstructions from this infected wood sliver. The longitudinal axis of the wood sliver is oriented vertically in Fig. 7 with the darker regions representing Mn and Fe ion concentrations in the wood cell walls in Fig. 7d and e, respectively. The highlighting of the wood tissue with Mn and Fe is in agreement with the increased amount of these ions in the cell walls seen from the 2-μm-thick sections cut from the same Fe-rich area (Fig. 3r and s, respectively). The brighter regions in the ion reconstructions are volumes of higher ion concentrations and likely hyphal fragments or dried ECM that remained attached to the wood sliver.

**Figure 8:**
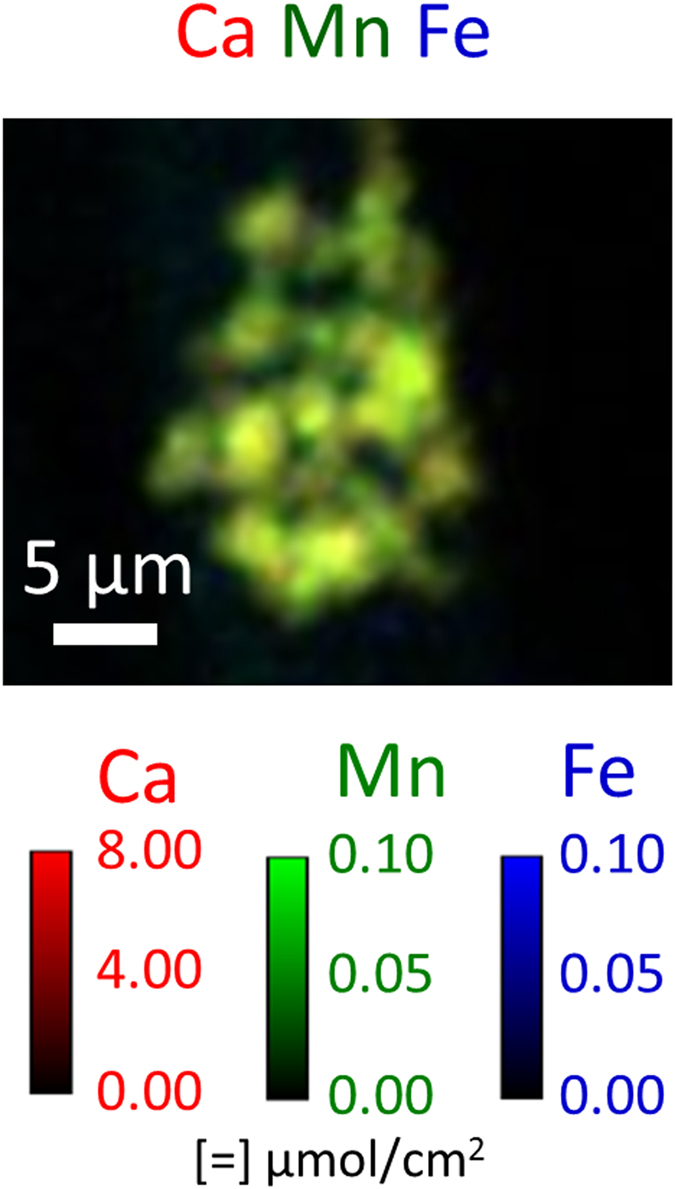
Ca Mn Fe 2D ion colocation map from an ion concentration of putative dried ECM attached near the bottom of the wood sliver (Fig. 7). Color intensities were scaled to optimize spatial visualization of ion distribution within the structure containing the hotspot, and not the ions inside the wood cell wall.

**Figure 9:**
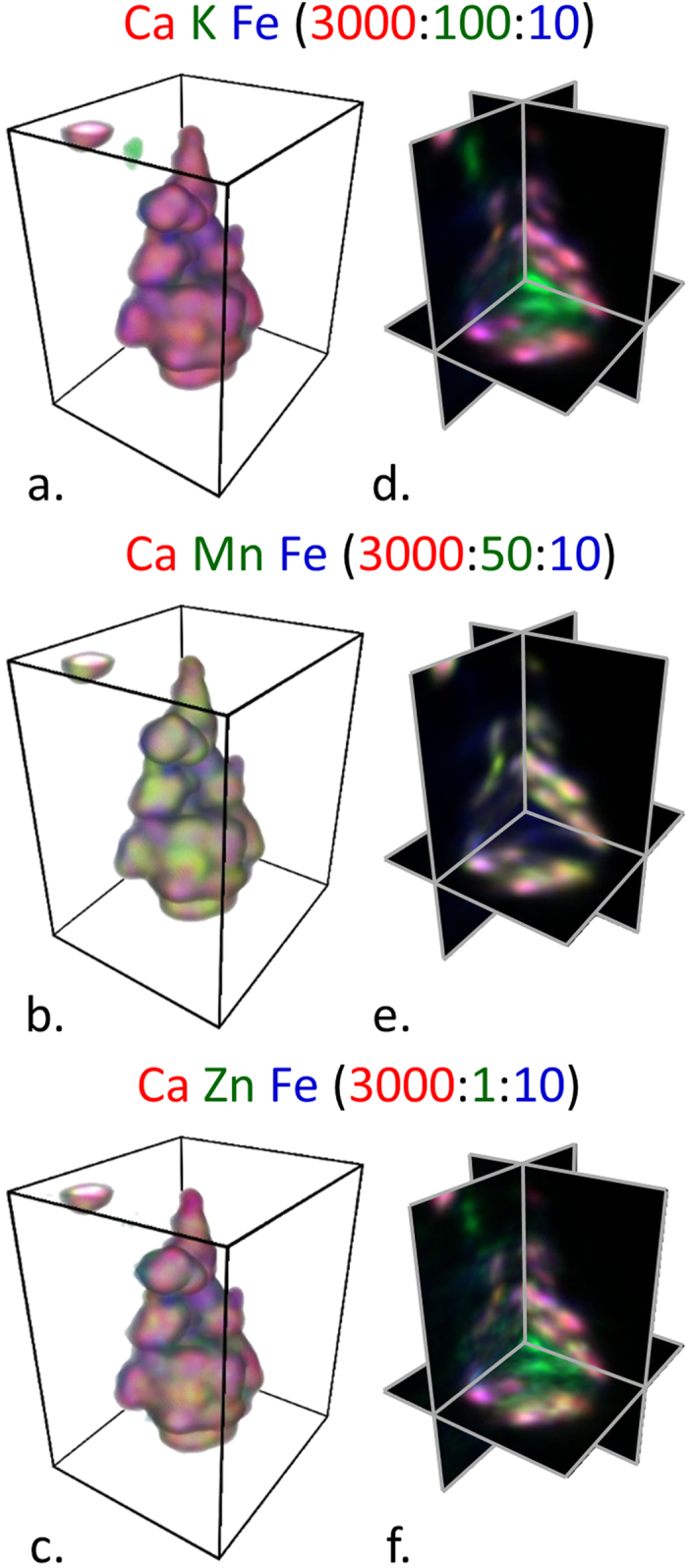
Ca K Fe (**a**,**d**), Ca Mn Fe (**b**,**e**), Ca Zn Fe (**c**,**f**) ion colocation volume reconstructions (**a**–**c**) and orthogonal cuts (**d**–**f**) from the volume of ion concentration within ECM residual material located near the bottom of the wood sliver (Fig. 7). The boxes have a rectangular base of 21 μm by 24.3 μm and a 30 μm height. The ratios indicated in the figure are molar ratios with respect to the amount of Zn. See Supplementary Movie 2 for 360-degree rotations of volumes and orthogonal views.

Although long intact filamentous hyphae like those in Fig. 4g–l were not observed in 3D within the sliver, the largest ion concentration near the bottom of the wood sliver was chosen for additional study. A 2D Ca Mn Fe colocation map of this region is shown in Fig. 8 and it had a similar composition and structure to the putative dried ECM shown in Fig. 6c. Therefore, this volume is also likely an ECM residue. Ion colocation volume reconstructions and orthogonal views were constructed (Fig. 9 and Supplementary Movie 2) and regions of high Ca concentrations were observed which may indicate early stages of Ca-oxalate crystals forming. Prior to drying of the section, the Ca concentrations would have been more broadly distributed along the fungal hyphae. The colocations also show an inhomogeneous distribution of K, Ca, Mn, Fe, and Zn within the ECM residue. Transition metals are known to play an important role in the mechanisms of fungal degradation, and particularly in brown rot fungal deconstruction of wood cell walls. For example, understanding how iron is transported and where it is deposited by fungi helps to support our understanding of iron redox-cycling associated with CMF oxygen radical generation. Further, knowledge of calcium deposition enhances our understanding of how oxalate is solubilized by the fungus, potentially in an effort to control pH at the micro-scale. Equally important are our observations of ions that are mobilized to a lesser extent, which suggests lesser participation these ions in brown rot decay mechanisms. Future research will enhance our understanding of the movement of ions, and the redox state to help better understand non-enzymatic and enzymatic oxidative deconstruction mechanisms.

## Discussion

XFM was shown to be useful in visualizing ions involved in the fungal degradation of wood across multiple length scales down to the submicron resolution needed to map ions in individual wood cell walls and fungal hyphae. Differential translocation of the ions prevalent in the fungal hyphae was observed indicating that fungi solubilize and transport ions from the soil to wood, and both into and out of the wood cell walls. The reasons for translocation and solubilization of some ions is not known, but for the oxidized forms of Fe and Mn it is well known that oxalate sequesters and solubilizes these ions, permitting mobilization and functional access to the organism^2^. Because of the increase in Fe during decay and the spatial control at both the bulk (Fig. 1k) and cellular (Fig. 3s) length scales, mobilization is likely related specifically to the degradation of the wood cell wall. Our findings suggest that an iron exchange for calcium associated with oxalate surrounding the fungal hyphae in the wood cell lumen, as part of the CMF mechanism for degrading the wood cell wall, may drive this mobilization. For ions such as K, mobilization likely relates directly to fungal physiological requirements and moisture within the decaying wood. For the other ions studied, other physiological requirements may require their mobilization.

Comparisons of XFM ion maps between the wood blocks (Fig. 1), 2-μm-thick sections cut from the test blocks (Fig. 3), and hyphae (Fig. 4) provide new insights into the location and movement of ions between the wood and colonizing fungus. For instance, Fig. 1k shows an overall increase of Fe in the decay block. However, from the large field-of-view maps alone (Fig. 1) it could not be determined if the Fe is in the fungal hyphae or the wood cell walls. The submicron resolution maps of the 2-μm-thick transverse sections cut from exposed Block 3 (Fig. 3m and s) were needed to clearly show that Fe had diffused into the wood cell walls. Furthermore, the Fe map of the hyphae (Fig. 4k) suggested that any hyphae colonizing the lumina in the exposed blocks would also contain Fe and would be contributing to the Fe in Fig. 1k. The increase in Fe after exposure to *Serpula lacrymans* as observed visually in the XFM maps (Figs 1, 3 and 4) and quantitatively in the wood cell walls (Table 1) was generally consistent with results from previous studies that used conventional inductively coupled plasma analyses to assess bulk changes in ion concentrations wood undergoing brown rot attack^8^,^9^,^35^. Overall Ca intensity was also observed to increase in the large field-of-view XFM maps of the wood blocks (Fig. 1), consistent with prior bulk inductively coupled plasma measurements, although the XFM measurements of Ca concentrations specifically within the wood cell walls (Figs 3 and 4, Table 1) decreased after decay. Our results demonstrate how XFM may allow the quantification and mapping of ions inside of wood cell walls, and in association with specific fungal hyphae, which should provide new insights into how the ions are utilized in the degradation processes inside the cell walls.

The XFM in this study was performed under dry conditions. The drying process may have modified ion locations in the fungal hyphae, but not likely in the wood. Ions are known not to diffuse through wood under dry conditions^27^, and therefore the inhomogeneous distributions observed here in the decayed wood at the block level (Fig. 1g–l) and cellular level (Fig. 3q–t) were likely representative of the ion distribution in wood during decay. Additionally, it should be noted that during drying the dimensions of wood blocks and cell walls may shrink by as much as 10% compared to their hydrated dimensions. Although drying the samples may have preserved the location of ions within the wood and wood cell walls, the distributions of ions in the hyphae - especially in the gelatinous ECM - likely moved during the drying process and the ECM appears to have agglomerated during drying, which may have resulted in areas of concentrated ions. Therefore, although the amount of ions in the hyphae and ECM would not be expected to change much during drying, the spatial distributions observed in the hyphae and dried ECM was likely not representative of the ion spatial distributions in living fungi. In the future, following previous work using XFM to map ions in hydrated plant tissues^37^,^38^ and using the methods described in this paper in combination with an *in situ* humidity-controlled chamber^27^, we will use XFM to map ions in hydrated live fungal hyphae and wood cell walls during decay over a time course. This should give us more information about ions in live hyphae and wood cell walls over time as an active fungus decays wood.

## Conclusions

The results of these experiments show synchrotron-based XFM to be a powerful multiscale tool for simultaneously mapping numerous ions in complex substrates, such as decaying wood. The XFM ion maps showed fungi actively control the distribution and amount of ions during decay. In contrast to conventional bulk measurement of ions in decayed wood blocks, XFM maps allowed study of both the quantification and spatial distribution of ions in wood during decay. A substantial increase in Fe concentration in both the wood block and wood cell walls was observed, consistent with current theory that brown rot progression is mediated by Fe redox reactions^15^. XFM results also suggested the movement of Ca out of the wood cell walls and into the area occupied by adjacent fungal mycelium, and this we propose also may be required to satisfy the release of Fe from oxalate as part of the CMF mechanism. Future studies will focus on improving these methods and on using decayed samples within a humidity controlled environment to permit imaging of a living fungus as it colonizes wood cell walls and initiates degradation.

## Methods

### Soil chamber methods

Wood samples were exposed to the wood-decay fungus using a modified AWPA E10 soil-block assay^39^. The soil and southern pine (*Pinus* sp.) feeder strips were first autoclaved in the glass containers. For inoculation, agar plugs taken from the leading edge of an active culture of *Serpula lacrymans* (Wulfen) P. Karst (ATCC36325) were used to inoculate both end grain surfaces of the feeder strip. The fungus was allowed to colonize the feeder strip for two weeks to ensure adequate mycelial coverage of the feeder strip. Two types of southern pine wood specimens were then prepared for decay experiments. One type of specimen was blocks of wood, 10 mm on a side, which were prepared by sawing, sterilized by autoclaving, and then placed on the colonized feeder strips for a prescribed period of time. After exposure, the 10 mm blocks were removed from the soil chamber and air-dried. The other type of specimen was 2 μm-thick tangential-longitudinal sections of latewood that were prepared using a Leica EM UC7 ultramicrotome (Wetzlar, Germany). Rectangular sections 100–300 μm in the tangential direction and 2–3 mm in the longitudinal direction were cut into a diamond knife boat filled with deionized water. Individual sections were lifted from the water and held flat while drying. Small frames were made of 0.13 mm-thick Kapton^TM^ (DuPont, Wilmington, Delaware, USA) film and section ends were secured to the frames using small pieces of Kapton^TM^ tape such that the section freely spanned a 1-mm-wide hole in the center of the frame. The sections were then exposed to active *Serpula lacrymans* cultures by placing the Kapton^TM^ frames with attached sections on top of the colonized feeder strips in the soil-block assays for prescribed periods of time. After exposure, the Kapton^TM^ frames with attached sections were removed from the soil chamber and air-dried.

### X-ray fluorescence microscopy (XFM)

Large field-of-view XFM was performed on the 8-BM beamline at the Argonne National Laboratory (Argonne, IL, USA) Advanced Photon Source (APS) to map ions in the 10 mm wood blocks (Figs 1 and 2). Specimens for the XFM mapping were prepared by cleaving approximately 2-mm-thick sections from the center of the dried blocks along the longitudinal direction. The sections were mounted between layers of 4-μm-thick 3525 Ultralene film (SPEX SamplePrep, Metuchen, NJ, USA). The incident X-ray beam energy was 10.1 keV and the spot size was approximately 30 μm wide by 20 μm high. Elemental maps were built by raster-scanning imaging, using 25-μm step sizes with 100-ms dwell times at each step.

Submicron resolution XFM was performed on three different types of samples using the APS beamline 2-ID-E (Figs 3, 4, 5, 6, 7 and 8). The incident X-ray beam energy was 10.2 keV and spot size was approximately 0.8 μm in the horizontal and 0.5 μm in the vertical. Elemental maps were built in 0.3-μm step sizes with 5-ms dwell times at each step. The samples analyzed by submicron resolution XFM were:

1. Two-μm-thick transverse sections that were cut from the 10 mm wood block sections mapped with the large field-of-view XFM. These sections were sectioned and collected dry to prevent ion movement in the cell walls using a diamond knife fit into the ultramicrotome under ambient conditions. The sections were secured between two Norcada 200-nm–thick silicon nitride windows (Edmonton, AB, Canada).
2. Two-μm-thick tangential-longitudinal sections attached to a Kapton^TM^ frame that were directly exposed to the colonized feeder strips in the soil-block assays as described previously.
3. A wood sliver approximately one tracheid across and a few mm long, which was excised manually using a hand razor under a dissecting microscope from the 10 mm wood block section that was mapped with the large field-of-view XFM. The sliver was used in an XFM tomography experiment and the tomography data set consisted of 60 projections that were collected with 3° of rotation between each projection.

For all XFM experiments, data analysis was performed using MAPS software^40^. In brief, the full spectra were fit to modified Gaussian peaks, the background was iteratively calculated and subtracted, and the results were compared to standard reference materials (RF8-200-S2453, AXO GmbH, Dresden).

#### Tomography reconstruction

Image registration and tomographic reconstruction were performed using MAPSToTomoPy, a program written in Python programming language^41^. MAPSToTomoPy takes the fully analyzed data from MAPS software, preprocesses the data using functions such as normalization, glitch removal, and 2D projection registration, and calls on TomoPy^42^ to carry out 3D reconstruction for each of the chemical elements. An iterative reconstruction algorithm, MLEM, with 7 iterations was used in this particular case.

#### Image analysis and visualization

Image analysis and presentation were carried out by ImageJ^43^ using 32-bit tiff images exported from the MAPS software for 2D images and 32-bit tiff image stacks from MAPSToTomoPy for 3D images. Mass concentrations in two-dimensional images were converted to molar concentrations by dividing by the element’s molar mass. No filtering was performed on the large field-of-view XFM images (Figs 1 and 2). A Gaussian blur (sigma (radius) = 1) was applied to all submicron resolution XFM two- and three-dimensional images (Figs 3, 4, 5, 6, 7 and 8). Three-dimensional volume reconstructions (Figs 7 and 9; Supplemental Movies 1 and 2) and orthogonal slices (Fig. 9) were created using the 3D Viewer ImageJ plugin^44^.

## Additional Information

**How to cite this article**: Kirker, G. *et al*. Synchrotron-based X-ray fluorescence microscopy enables multiscale spatial visualization of ions involved in fungal lignocellulose deconstruction. *Sci. Rep.* **7**, 41798; doi: 10.1038/srep41798 (2017).

**Publisher's note:** Springer Nature remains neutral with regard to jurisdictional claims in published maps and institutional affiliations.

## Supplementary Material

Supplementary Information

Supplementary Movie 1

Supplementary Movie 2

## Figures and Tables

**Table 1:** Calculated average ion concentrations of the undecayed and decayed wood cell walls shown in Fig. 3.

Element	Undecayed	Decayed (Fe-gradient)	Decayed (Fe-rich)
	(μmoles/g cell wall)	(μmoles/g cell wall)	(μmoles/g cell wall)
K	25 ± 12	19 ± 11	20 ± 11
Ca	21 ± 17	4 ± 6	10 ± 9
Mn	4 ± 3	3 ± 2	6 ± 4
Fe	0.7 ± 0.9	3 ± 2	7 ± 4
Zn	1.4 ± 0.5	1.5 ± 0.6	1.5 ± 0.6

The intensities in Fig. 3 were converted to μmoles/g cell wall by dividing by the 2 μm cell wall thickness and then dividing by an assumed 1.5 g/cm^3^ cell wall density. Masks were created to exclude the cell wall lumina and K and Ca hotspots in the decayed wood that were assumed to be fungal hyphae fragments with associated ECM. The average concentrations of the pixels in the remaining areas were calculated to quantify the cell wall ion concentrations. The reported uncertainty is the standard deviation.
