# Supplementary material for: Synchrotron-based X-ray fluorescence microscopy enables multiscale spatial visualization of ions involved in fungal lignocellulose deconstruction
Source: Sci Rep. 2017 Jan 31;7:41798. doi: 10.1038/srep41798 (PMC5282557; doi:10.1038/srep41798)
Supplement: Supplementary Information [file srep41798-s1.pdf]

Synchrotron-based X-ray fluorescence microscopy enables multiscale spatial visualization of ions involved in fungal lignocellulose deconstruction

Authors: Grant Kirker[1], Sam Zelinka[2], Sophie-Charlotte Gleber[3], David Vine[3], Lydia Finney[3], Si Chen[3], Young Pyo Hong[4], Omar Uyarte[5,6,7], Stefan Vogt[3], Jody Jellison[8], Barry Goodell[5], Joseph E. Jakes[9]*

[1]Durability and Wood Protection Research, USDA Forest Service, Forest Products Laboratory, Madison, WI, USA.

[2]Building and Fire Sciences, USDA Forest Service, Forest Products Laboratory, Madison, WI, USA.

[3]Advanced Photon Source, Argonne National Laboratory, Argonne, IL, USA.

[4]Department of Physics and Astronomy, Northwestern University, Evanston, IL, USA.

[5]Department of Sustainable Biomaterials, Virginia Tech, Blacksburg, VA, USA.

[6]Departamento de Biotecnologia, Escola de Engenharia de Lorena, Universidade de São Paulo, Brazil.

[7]Centro I+D, CMPC Celulosa, Chile.

[8]Center for Agriculture, Food and the Environment, University of Massachusetts - Amherst, Amherst, MA, USA.

[9]Forest Biopolymers Science and Engineering, USDA Forest Service, Forest Products Laboratory, Madison, WI, USA.

*Correspondence to jjakes@fs.fs.us

Supplementary Movie 1. 360 degree rotations micron-scale resolution XFM ion volume reconstructions of a wood sliver and attached fungal fragments and ECM residue cut from the Fe-rich region near the bottom of exposed Block 3 (Figure 1). During tomographic reconstruction, the quantification of the ions was lost, however relative ion concentration differences can be detected and compared. The ratios indicated are molar ratios with respect to the amount of Zn. The boxes have a square base of 90 µm per side and a 75 µm height.

Supplementary Movie 2. 360-degree rotations of Ca K Fe (a, d), Ca Mn Fe (b, e), Ca Zn Fe (c, f) ion colocation volume reconstructions (a-c) and orthogonal cuts (d-f) from the volume of ion concentration

within ECM residual material located near the bottom of the wood sliver (Figure 7). The boxes have a rectangular base of 21 µm by 24.3 µm and a 30 µm height. The ratios indicated in the figure are molar ratios with respect to the amount of Zn.
